# Cartilage Integrity: A Review of Mechanical and Frictional Properties and Repair Approaches in Osteoarthritis

**DOI:** 10.3390/healthcare12161648

**Published:** 2024-08-19

**Authors:** Przemysław Krakowski, Adrian Rejniak, Jakub Sobczyk, Robert Karpiński

**Affiliations:** 1 Department of Trauma Surgery and Emergency Medicine, Medical University, 20-059 Lublin, Poland; 2 Orthopaedic and Sports Traumatology Department, Carolina Medical Center, Pory 78, 02-757 Warsaw, Poland; adrian.rejniak@carolina.pl (A.R.); jakub.sobczyk@carolina.pl (J.S.); 3 Department of Machine Design and Mechatronics, Faculty of Mechanical Engineering, University of Technology, 20-618 Lublin, Poland; 4 Department of Psychiatry, Psychotherapy and Early Intervention, Medical University, 20-059 Lublin, Poland

**Keywords:** cartilage, osteoarthritis, cartilage friction, cartilage biomechanics, friction, wear

## Abstract

Osteoarthritis (OA) is one of the most common causes of disability around the globe, especially in aging populations. The main symptoms of OA are pain and loss of motion and function of the affected joint. Hyaline cartilage has limited ability for regeneration due to its avascularity, lack of nerve endings, and very slow metabolism. Total joint replacement (TJR) has to date been used as the treatment of end-stage disease. Various joint-sparing alternatives, including conservative and surgical treatment, have been proposed in the literature; however, no treatment to date has been fully successful in restoring hyaline cartilage. The mechanical and frictional properties of the cartilage are of paramount importance in terms of cartilage resistance to continuous loading. OA causes numerous changes in the macro- and microstructure of cartilage, affecting its mechanical properties. Increased friction and reduced load-bearing capability of the cartilage accelerate further degradation of tissue by exerting increased loads on the healthy surrounding tissues. Cartilage repair techniques aim to restore function and reduce pain in the affected joint. Numerous studies have investigated the biological aspects of OA progression and cartilage repair techniques. However, the mechanical properties of cartilage repair techniques are of vital importance and must be addressed too. This review, therefore, addresses the mechanical and frictional properties of articular cartilage and its changes during OA, and it summarizes the mechanical outcomes of cartilage repair techniques.

## 1. Introduction

Osteoarthritis (OA) is one of the main causes of disability around the world, especially among the elderly population. Although OA is considered an age-related disease, studies have shown that osteoarthritic changes can be found in a younger population [1,2,3]. The global burden of disease report estimates that over 527 million people suffer from OA [4,5]. The development of OA can be triggered by many different factors, including obesity, sports, genetic factors, previous injuries, work environment or joint anatomy abnormalities [6,7,8]. Osteoarthritis is also an important socioeconomic problem. It is estimated that the costs of OA can reach up to 2.5% of the gross domestic product [9]. With the aging of the population, an increase in OA prevalence is expected [10]. Articular cartilage (AC), which is a highly specialized tissue producing smooth, painless, and almost frictionless movement, is most significantly affected during OA progression. Moreover, cartilage presents very limited repair capacity [11,12,13,14]. Once the cartilage structure is compromised, osteoarthritic degeneration begins, leading to joint failure and pain as an end result [11,15]. The inevitable degradation of cartilage starts from its superficial layer [16], which is also the most important layer in preserving tribological properties due to its collagen orientation and composition. The surface roughness of healthy articular cartilage ranges from 1 nm to 150 nm, depending on the joint [17]. Interestingly, the surface roughness of total joint replacements typically range from 40 nm to 200 nm [18]. Increased friction during OA progression as well as loads exerted on cartilage induce secretion of proinflammatory cytokines such as IL-1, which further increases surface roughness and friction [19,20]. Modern orthopedics considers cartilage as the most important structure to be preserved and protected in diarthrodial joints in order to reduce OA progression. Various joint-sparing treatments have been proposed in the literature, from conservative [21,22,23,24] up to different surgical treatment options, including microfractures, load-shifting osteotomies, various scaffold options, as well as cartilage culture and implantation techniques [25,26,27,28]. Clinical outcomes of those procedures have been extensively studied by orthopedic surgeons and most commonly survivor time (time when total joint replacement is required) is one the most important outcome measures. Nevertheless, no strict protocol for cartilage repair and regeneration has been established to date. To better understand the nature of cartilage regeneration techniques, orthopedic surgeons should understand the biomechanical and tribological properties of cartilage and its repair techniques. This review summarizes the anatomy and mechanical properties of healthy cartilage, as well as cartilage repair techniques.

## 2. Healthy Cartilage—Anatomy, Mechanical Properties, Synovial Fluid

The cartilage is a viscoelastic type of connective tissue, originating during the embryonic phase of human development, prior to the onset of osteogenesis [29]. Its mean thickness was estimated at 1.4 mm. However, it is worth noting that the study was performed on an elderly population, in which cartilage loss is to be expected. A more recent study by Guo et al., who analyzed a total of 700 MRI results, has found that articular cartilage thickness ranges from 1.79 mm to 3.13 mm depending on location [30].

The primary role of articular cartilage is to create a smooth, lubricated overlay for low-friction articulation and to allow loads to be transmitted to the underlying subchondral bone. The unique material properties of cartilage allow it to withstand strong contact forces while dispersing the ensuing compressive stimulus to the subchondral bone underneath. Hyaline cartilage consists of chondrocytes and an extracellular matrix (ECM). Chondrocytes are cells that exhibit a high degree of specialization and metabolic activity, performing a distinct function in the processes of extracellular matrix creation, maintenance and repair. They are characterized by low apoptotic activity and an inability to divide [31].

Chondrocytes derive from mesenchymal stromal cells and comprise around 2% of the overall volume of the articular cartilage [32]. Chondrocytes rarely form intercellular connections for signal transduction and transmission. However, they respond to growth factors, mechanical stresses, piezoelectric forces, and hydrostatic pressures. Schätti et al. showed that bone marrow mesenchymal stromal cells show upregulation in chondrocyte-specific gene expression when biaxial loading is applied [33]. Chondrocytes also affected by compression frequency. Sah et al. [34] showed that cyclic loading with a frequency of 0.1 to 1 Hz stimulates collagen and proteoglycans synthesis, while, on the other hand, static loading was related to dose-dependent ECM degradation [35]. Unfortunately, chondrocytes have a limited mitotic capacity, which, in turn, decreases the intrinsic capacity of cartilage for healing after injury [36,37].

The biochemical composition of cartilage includes water, collagen, and proteoglycans [36]. Cartilage is a type of tissue that exhibits anisotropic and viscoelastic features, enabling it to withstand compressive, tensile, and shear forces. The compressive strength of tissue is attributed to the presence of water and proteoglycans. This phenomenon is attributed to negative electrostatic repulsion forces [36,38]. Under compression, negative charges of proteoglycans are pushed closer together and, as a result, the repulsive force increases, adding significantly to cartilage stiffness [36,39,40]. The resistance to tensile stresses is mostly conferred by collagen fibrils. This is based on the mesh structure of collagen fibrils interconnections [41] and its unique arrangement depending on the cartilage layer. Hyaluronic acid, lubricin, and matrix permeability play a crucial role in reducing friction on the joint surface. Decorin, a tiny leucine-rich proteoglycan, regulates the micromechanics and mechanobiology of the cartilage pericellular matrix. In vivo, as chondrocytes reside in an aggrecan-rich, highly negatively charged osmomicroenvironment, decorin regulates chondrocyte mechanotransduction primarily via controlling the integrity of aggrecan in the PCM [42].

The surface of cartilage is enveloped by a delicate layer known as lamina splendens [30,43]. This layer is composed of proteins and exhibits an acellular and non-fibrous nature. Its thickness varies in the range from a few hundred nanometers to one micrometer. The formation of lamina splendens has been postulated to occur through a gradual build-up of proteins originating from synovial fluid. It provides a low-friction interface for cartilage and plays a key role in responding to mechanical loads [44]. It is also the first cartilage layer to degrade during the progression of OA [45]. However, chondrocyte implantation, for instance, does not regenerate lamina splendens [46].

The superficial zone lies beneath lamina splendens. It accounts for 10–20% of the cartilage thickness. The collagen fibers in this zone have relatively small diameters (18 ± 5 nm) and are packed tightly, running parallel to the surface [47]. This particular architectural arrangement is well-suited for effective dispersing and mitigating the impact of substantial shear stresses [48]. The proteoglycan concentration within this layer is comparatively low, resulting in its higher permeability when compared to other layers of cartilage [49,50,51]. And what is most interesting, permeability rather than layer thickness is responsible for dynamic friction in contrast to start-up friction, which is dependent on the layer thickness [52]. The chondrocytes located in the superficial zone of cartilage are in control of the secretion of lubricating proteins such as superficial zone proteins (SZPs) [53] along with collagen I [54]. These proteins are exclusive to the superficial zone, absent in the other layers of the cartilage [55].

The middle zone, which comprises 40–60% of the cartilage thickness, is distinguished by a reduced cellular concentration together with the presence of spherical chondrocytes. Type II collagen is the predominant ECM component inside this region [56]. The structure is composed of arcades that are interconnected by small-scale fibers with random orientations [48,57]. The middle zone exhibits the most elevated concentration of proteoglycans compared to other zones [49,50,51]. The chondrocytes located in the middle layer demonstrate a substantial expression of collagen II and proteoglycans, including aggrecan [56].

The region referred to as the deep zone, along with the calcified zone, which comprises 20–50% of the cross-sectional length, exhibits a cellular and collagen fiber organization that is oriented perpendicular to the subchondral bone. While the concentration of proteoglycans may rise, the cellular density in the deep zone is significantly lower in contrast to the middle and superficial zones [50,51]. Chondrocytes in this layer demonstrate a reduced capacity for synthesizing and secreting collagen II [58]. Collagen X secretion can also be observed in the deep zone, where it contributes to structural integrity and shock absorbance [59].

The deep and calcified zones are separated by a narrow line known as the “tidemark”. The tidemark acts as a means of anchoring more pliable and vertically aligned collagen fibrils found in the innermost region of non-calcified articular cartilage. This anchoring mechanism is considered to help protect these fibrils from being disrupted or torn at their attachment point to the calcified zone [60].

**Figure 1:**
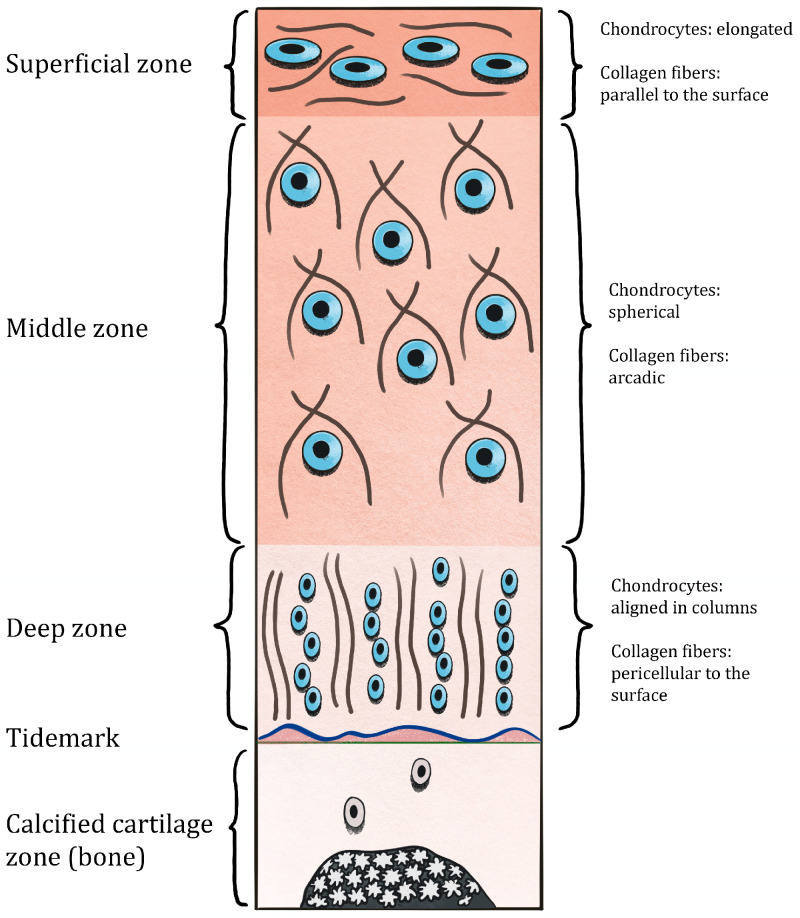
Cartilage layers illustrating differences in collagen mesh structure and chondrocyte arrangement.

The calcified zone is composed of hydroxyapatite, which serves as a barrier between the rigid bone and the pliable cartilage, reducing a mechanical gradient [61]. The layered architecture of cartilage is presented in Figure 1.

**Figure 2:**
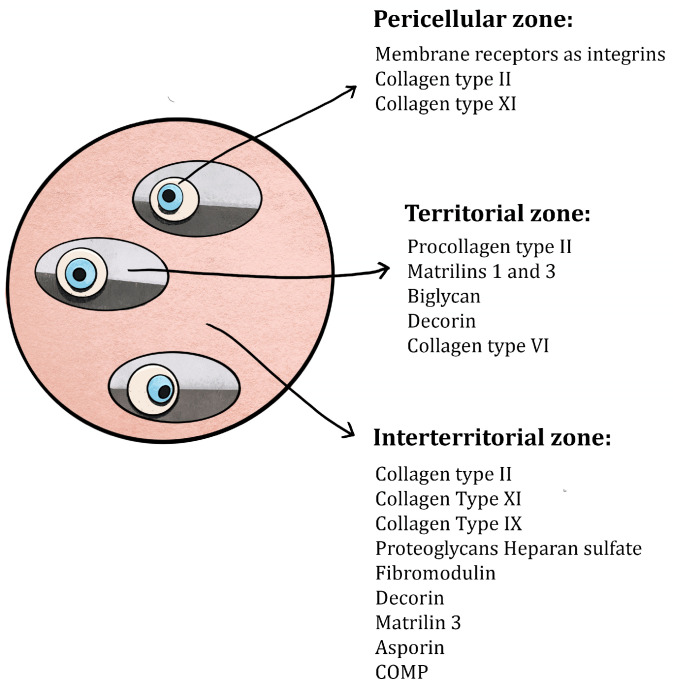
Cross-sectional view of ECM, with a division into pericellular, territorial, and interterritorial cartilage sections.

In addition to the presence of zone-related differences in the structure and composition of cartilage, the matrix is composed of numerous specified regions that are delineated by factors such as closeness to chondrocytes, composition, and collagen content. The ECM is divided into the pericellular, territorial, and interterritorial regions [36], as shown in Figure 2. In this context, it can be observed that each chondrocyte is enveloped by a translucent pericellular glycocalyx matrix (Pg) and is also enclosed by a pericellular capsule (Pc). This structure is known as the chondron [62]. A combination of these two constituents is commonly known as the pericellular matrix. The pericellular capsule is enveloped by a territorial matrix (Tm) and an interterritorial matrix (Im), which is sometimes referred to as ECM [63]. A notable presence of aggrecan, link protein, and hyaluronan can be observed inside a pericellular matrix (Pg and Pc) [64]. It can, however, be noticed that these macromolecules are in a dissociated state and that the aggrecan–hyaluronan complex has not yet been formed. The collagen composition exhibits variations in comparison to the bulk or interterritorial matrix (Im) of cartilage [65]. Collagen II is observed in the form of slender strands, with their diameters ranging from 10 to 15 nanometers, which are intricately intertwined to create a compact and densely woven arrangement known as the pericellular capsule [64]. This architectural structure is believed to function as a hydrodynamic mechanism for safeguarding chondrocytes during loading. It acts as a pliable cushion that is capable of supporting load by reversible deformation depending on the amount of stress [66]. According to Chandrasekaran et al., SEM scans revealed intricate collagen fibril structures on the mandibular condyle cartilage and articular disk. The fibrillar diameter on the condyle cartilage surface was 22.3 ± 0.3 nm (mean ± 95% CI from 300 fibrils measured on n = 3 animals), significantly thinner (*p* < 0.0001) than those on the disk surfaces (33.4 ± 0.4 for superior surface, 32.9 ± 0.5 for inferior surface, both statistically comparable). Additionally, differences in fibril sizes were observed on the disk surfaces. The anterior end had the thickest fibrils (35.6 ± 1.1 nm), while the medial region had the thinnest (31.3 ± 0.8 nm). On the inferior side, the front end similarly had the thickest fibrils (34.0 ± 1.0 nm), and the central region had the thinnest (31.2 ± 0.9 nm) [67]. Collagen types IX and VI, along with collagen II, present increased quantities within the pericellular matrix, as opposed to the interterritorial matrix [62]. As previously elucidated, these collagens actively engage in cross-linking processes to contribute to the development of more substantial collagen II bundles. The composition of the territorial matrix (Tm) closely resembles that of the interterritorial matrix, except for the thinner collagen II fibrils and a larger assembly of proteoglycans, particularly chondroitin sulfate [68].

Collagen (75% of the dry weight) and proteoglycan (20–30% of the dry weight) are the primary load-carrying structural segments of the extracellular matrix, and their concentrations and architectures vary depending on the depth from the articular surface [69,70]. The following collagens types can be found in the hyaline cartilage: III (10%), IX (1%), XI (3%), as well as VI (1%, solely in the pericellular matrix surrounding chondrocytes) [71]. The surface zone has the maximum collagen concentration, with a 20% decrease in its concentration in the middle and deep zones. The content of proteoglycans is the lowest in the superficial zone and increases by up to 50% in the middle and deep zones [69,70]. The superior resistance to shear forces of the superficial zone among the four zones can be linked to the unique organization of collagen fibrils [72,73,74]. Moreover, the superficial zone protein, which is only produced by chondrocytes located in the superficial layer, further decreases friction and, therefore, protects cartilage against shear forces [75]. This is now recognized to be the very same molecule as the megakaryocyte-stimulating factor or lubricin [76]. Furthermore, it is synthesized by synovial cells. The concept is that lubricin plays a predominant role in supplying the almost frictionless articulation provided by the articular cartilage [76]. Collagen exhibits a limited elongation of less than 10% of its overall length, with a significant portion of this elongation attributed to the straightening of the fibers rather than to actual extension [77]. When all collagen molecules reach a straight configuration oriented perpendicular to the pulling axis and the entire potential for molecular straightening is used up, the collagen molecules themselves stretch, resulting in a markedly increased tangent microfibril stiffness at strains greater than 10% [78].

The articular cartilage is composed of two distinct phases: fluid and solid. The articular cartilage is primarily water, which constitutes up to 80% of its wet weight. About 30% of the water is located in the intrafibrillar region within the collagen structure, whereas a little proportion is located within the intracellular area. The residual fraction is confined to the interstitial voids of the matrix [79,80]. The tissue water contains dissolved inorganic ions, including sodium, calcium, chloride, and potassium [81]. The water concentration exhibits a gradual decrease from approximately 80% in the surface zone to 65% in the deep zone [82]. Its movement within cartilage and over the articular surface facilitates the transportation and dispersion of nutrients to chondrocytes, while also serving as a lubricant [36]. The interaction of proteoglycan aggregates and interstitial fluid results in negative electrostatic repulsion forces, providing articular cartilage with compressive resilience [36]. An instant application of articular contact forces during joint loading results in a rise in the interstitial fluid pressure. This causes the fluid to leak out of the ECM, creating a significant frictional drag on the matrix [83,84]. When the compressive force is released, the interstitial fluid returns to cartilage. The fluid cannot be easily squeezed out of the matrix due to the limited permeability of the articular cartilage [85]. The two opposing bones, along with the surrounding cartilage, serve to enclose and restrict the movement of the cartilage located beneath the contact surface. The purpose of these boundaries is to limit or control mechanical deformation.

Since the articular cartilage is naturally avascular, synovial fluid (SF) is an essential component of the biomechanical behavior, lubrication, and metabolism/nutrition of this tissue. The composition of SF, which is a dynamic reservoir of proteins produced from synovial and cartilage tissue, may serve as a biomarker for the health and pathophysiologic state of the joint. It has a consistency which can be compared to an egg white. Hyaluronic acid, lubricin (a protein from the superficial zone), and phospholipids at physiological amounts work in concert to enable SF to act as a boundary lubricant and reduce border friction in cartilage [86,87].

Glycosaminoglycans (GAGs), proteoglycans, and glycoproteins are some of the non-collagenous components of the ECM ground substance that are connected to the fibrillar components. GAGs are sugars composed of repeating disaccharide units that give rise to six primary subunits of articular cartilage: chondroitin sulfates 4 and 6, keratin sulfates, dermatan sulfate, heparan sulfate, and hyaluronan (or hyaluronic acid) [88]. They are negatively charged, repelling one another while drawing ions (such as Ca11 and Na1) and water to them. This ensures that their primary functional characteristics, i.e., water absorption and maintenance of the mechanical properties as well as ECM hydration, are maintained [77,89].

## 3. Factors Affecting the Mechanical Properties of Cartilage

The articular cartilage is responsible for load-bearing as well as facilitating translational and rotational movements inside the adjacent joint [90]. The biphasic composite material of articular cartilage is characterized by an intricate structure and composition, which allows it to effectively trap liquids while maintaining its fibrous and porous nature [91]. When an external force is exerted on the system such as the application of a load, the synovial fluid confined inside the system becomes pressurized. As a result, the pressurized synovial fluid squeezes through the surrounding tissue, developing frictional resistance against the solid matrix. This frictional drag facilitates the transfer of the applied load within the system [92]. The exceptional load-bearing properties of articular cartilage can primarily be attributed to load-transmission and fluid-pressurization mechanisms [93,94,95]. The material’s biphasic and viscoelastic characteristics have significant implications for its reaction to compressive, tensile, and shear loads. These responses are unevenly distributed across the depth of the mature cartilage [96]. Multiple evaluation methods have been implemented to study the mechanical properties of cartilage. The methods are described at the end of this Section.

### 3.1. Compressive Strength

The permeability and viscoelasticity combination is thought to be responsible for the compressive characteristics of cartilage. Due to its inherent traits, articular cartilage exhibits a non-linear reaction when subjected to mechanical forces, owing to its inhomogeneity, anisotropy, and poro-viscoelastic nature. The tissue has poor permeability, resulting in rapid pressurization of the interstitial fluid. The tissue’s hydraulic permeability and aggregate equilibrium compression modulus significantly depend on the water content and uronic acid concentration. This relationship serves as a physicochemical foundation for the observed reduction in the tissue’s permeability as compression increases [97,98,99,100]. The movement of interstitial water via the ECM leads to certain time-dependent reactions in the articular cartilage [101]. Tissue creep may require around 1000 s of load application to attain a condition of new equilibrium [101] and additional time for stress relaxation to achieve a state of equilibrium. It is worth mentioning that an increase in ECM deformation leads to a decrease in the average size of pores. Consequently, this results in an increased diffusional resistance between the interstitial water and the ECM [102]. The duration required to attain the equilibrium condition depends on the magnitude of load or displacement.

When an external force is applied, the fluid that was previously constrained within the tissue starts to move. Due to poor permeability of cartilage, this results in pressurization and generation of significant drag forces on the solid phase. These drag forces help to dissipate the stress [103]. When cartilage undergoes deformation, there is a drop in its porosity, resulting in reduced permeability. The values of permeability in cartilage normally fall within the range of 0.1 to 10 × 10^15^ m^2^/Pa s [94]. This means that the cartilage responds to external loads through the augmentation of hydraulic pressure and mechanical rigidity [99]. Joint cartilage compressive forces exhibit a fleeting nature in spite of their considerable magnitudes. These forces escalate from around 1–2 atmospheres during unloading [104] to a range of 100–200 atmospheres when an individual assumes an upright position. Furthermore, these forces cyclically fluctuate between 40 and 50 atmospheres [105]. The explanation for the compressive properties of cartilage has been traditionally grounded in the biphasic theory. This study identifies three primary forces: (a) a stress exerted by the solid phase, which is presumed to adhere to Hooke’s law and exhibit a linear stress–strain behavior; (b) a pressure generated by compression of the liquid phase, which, as previously mentioned, exhibits a time-dependent behavior; and (c) a friction generated between the liquid and solid phases. The fluid phase generates friction, which may be characterized using a linear formulation of Darcy’s law [106]. This friction depends on the permeability of the tissue and the pressure generated, both of which vary with time. Upon removal of the load, the tissue undergoes a process of regaining its initial shape. This recovery is facilitated by two mechanisms: the Donnan osmotic pressure effect [107], which redistributes the fluid inside the compressed region, and the presence of elastic qualities inherent in the solid phase [55]. The variability of the compressive characteristics is associated with the disparity in fluid flow. Consequently, the superficial zone, which exhibits a high level of permeability, is subjected to compressive forces reaching a maximum of 50%. The fluid flow experiences a significant reduction in the medium and deep zones, leading to compressive strains that are below 5% [108]. Cartilage deformation is prevented through its low permeability, which results in fluid pressurization, and the impermeability of the subchondral bone, which provides stability to the tissue. Throughout the course of the day, there are repeated instances of compression-relaxation events, resulting in strains of which 15–20% are irreversible. The original shape can be fully restored only after extended periods of rest [109].

### 3.2. Summary of Tensile, Shear, and Tribological Properties of Articular Cartilage

The tensile, shear, and tribological properties of articular cartilage constitute essential determinants of its mechanical behavior under both physiological and pathological conditions. These characteristics are of particular importance in the context of osteoarthritis (OA), wherein progressive structural and biochemical degradation impairs the tissue’s ability to distribute mechanical loads, increases interfacial friction, and reduces resistance to shear-induced deformation [110,111,112,113,114,115].

When subjected to compressive loading, articular cartilage exhibits tensile strain and viscoelastic responses, primarily governed by the intricate interactions between the collagen fiber network and the proteoglycan-rich extracellular matrix. Resistance to shear stress is predominantly provided by the solid matrix and is highly dependent on the zonal organization of collagen architecture and proteoglycan concentration. Both tensile and shear moduli vary as a function of cartilage depth, the methodology employed for mechanical evaluation, and the stage of degenerative change. On a macroscopic scale, these mechanical parameters correlate strongly with the orientation of collagen fibers and the tissue’s biochemical composition [116,117,118,119,120,121,122,123].

Tribological characteristics, including the coefficient of friction (COF), are influenced by several factors such as lubrication regime, the biochemical composition of synovial fluid, surface morphology, and the degree of tissue hydration. Healthy articular cartilage demonstrates remarkably low COF values (down to 0.002), primarily attributable to mechanisms of interstitial fluid pressurization and boundary lubrication mediated by molecular constituents such as lubricin and hyaluronic acid [124,125,126]. In osteoarthritic cartilage, the coefficient of friction is elevated, typically as a consequence of glycosaminoglycan depletion, surface fibrillation, and diminished synthesis of endogenous lubricants [127,128,129,130,131,132,133,134].

A wide array of experimental methodologies has been applied to characterize these biomechanical properties, including atomic force microscopy (AFM) [131,132,133], indentation testing [135,136,137,138], compression tests [139,140,141,142], tensile tests [143,144,145,146,147], and tribological evaluation. Each technique yields method-specific values, which may differ based on the measurement scale, applied loading conditions, and sample preparation protocols. While AFM enables nanoscale assessment of surface mechanical properties, macro-scale tribological tests are designed to replicate physiologically relevant joint loading and lubrication environments [148,149,150,151].

A comprehensive and detailed description of experimental methodologies, numerical findings, and interpretative frameworks related to cartilage mechanics is available in the review by Belluzzi et al. [152]. That work offers a rigorous analysis of depth-dependent variations in mechanical moduli, protocol-specific test outcomes, and limitations inherent to various measurement approaches, providing a valuable reference for readers seeking in-depth technical insight.

## 4. Mechanical and Tribological Changes Induced by Osteoarthritis

Osteoarthritis (OA) is a complex disorder that exhibits diverse clinical manifestations depending on its specific anatomical sites, natural progression, clinical subtypes, and different etiological variables. The articular cartilage within a healthy joint has the capacity to endure substantial forces that arise from weight-bearing and joint movement throughout an individual’s lifespan. A hypothesis was formulated that persistent excessive stress and compromised biomechanical factors had detrimental effects on the joint, ultimately leading to the degradation of articular cartilage and a subsequent inflammatory response. Consequently, these symptoms later resulted in stiffness, edema, and reduced mobility. The current understanding is that osteoarthritis is a multifaceted process involving several inflammatory and metabolic variables [153,154].

### 4.1. Molecular Changes

During the early phases of osteoarthritis (OA), chondrocytes exhibit limited capacity for effective restoration of the damaged matrix. This is mostly due to an increasing activity of catabolic cytokines and matrix-degrading enzymes which hinder the repair process [155]. Unfortunately, this initiates the release of proteoglycans and the degradation of type II collagen on the cartilage surface. Subsequently, an elevation in water levels occurs, which is linked to the depletion of negatively charged glycosaminoglycans. This depletion subsequently leads to matrix swelling [156,157,158].

The breakdown of the cartilage matrix begins in the surface zone of cartilage and then expands into further zones as OA advances [159]. This phenomenon is correlated with a significant decline in the tensile strength of the extracellular matrix [160]. The breakdown of collagen and proteoglycan molecules, which are subsequently internalized by synovial macrophages, elicits the secretion of proinflammatory cytokines such as TNFα, IL-1, and IL-6. The binding of these cytokines to the receptors on chondrocytes results in a subsequent release of metalloproteinases and a suppression of type II collagen synthesis, thereby promoting the breakdown of cartilage [161]. Il-1β is considered a fundamental cytokine for OA progression. This cytokine not only induces secretion of proteases but also inhibits key type II collagen synthesis by osteoblasts [162]. This mechanism is supported by TNF-α [163] and Il-6 [161].

Alterations in PCM micromechanobiology are among the earliest signs of OA onset. Aggravated chondrocyte catabolism causes local degradation of proteoglycans, particularly aggrecan, in the PCM, resulting in worse micromechanical characteristics. This disrupts chondrocytes’ normal mechanosensing, contributing to the vicious cycle of cartilage breakdown in OA. The local PCM micromodulus (Eind, PCM) and mechanically induced chondrocyte [Ca^2+^]i activity are two crucial early indications of PTOA onset. Attenuating PCM degradation can protect chondrocyte mechanosensing, potentially protecting joint health, as local alterations occur before larger matrix changes. Exploring cell-ECM mechano-crosstalk at the nm-to-µm scale provides a basis for creating novel ways for early PTOA identification or treatments by targeting cartilage PCM [164]. These findings may also have implications for other load-bearing illnesses.

Multiple proteases have been described as OA triggers in the literature, out of which the most important are MMP-1, -3, -9, and 13 [165]. What is worth noting is the fact that degraded ECM components are a stimuli for further inflammatory response, which, as a result, progresses the OA. Antibodies directed against ECM proteins can be found in serum samples from patients with osteoarthritis and rheumatoid arthritis [166,167].

Apart from cartilage degradation and inflammatory activation, gross remodeling of subchondral bone is also present and proposed as a trigger towards further cartilage degradation [168]. Platelet-derived growth factor (PDGF) elevated levels in subchondral bone promote vessel formation and, therefore, progression of OA [169]. Another cytokine secreted by osteoblasts in subchondral bones is prostaglandin E2 [170], which has a detrimental effect on cartilage mostly by increasing the production of MMPs.

### 4.2. Structural Changes

**Figure 3:**
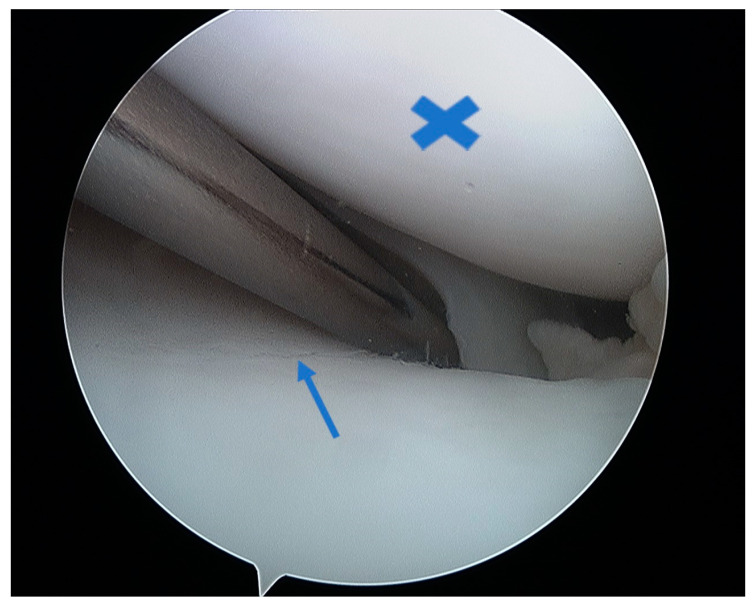
Arthroscopic view of healthy cartilage on the medial femoral condyle (asterisk) and grade I lesion on the medial tibial condyle with visible superficial layer fibrillation (arrow).

**Figure 4:**
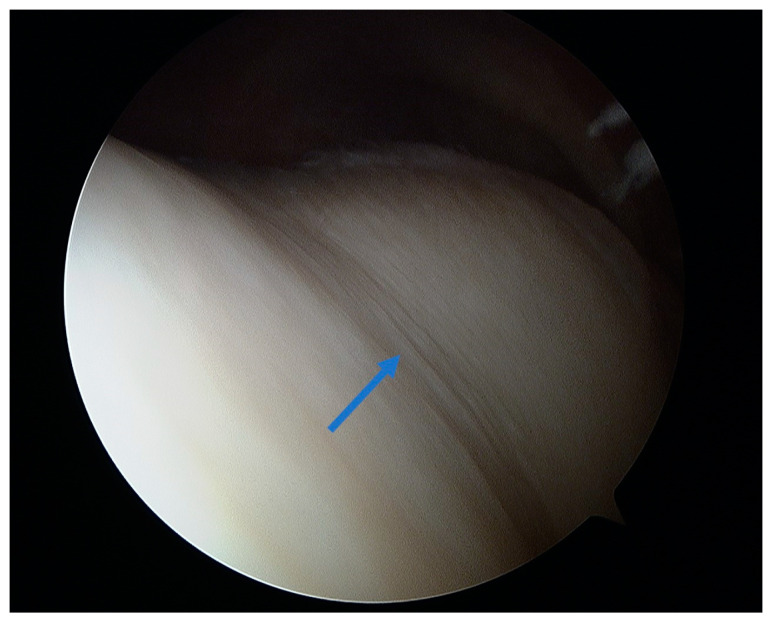
Arthroscopic view of grade II lesion on the femoral trochlear groove with visible longitudinal fissures (arrow).

**Figure 5:**
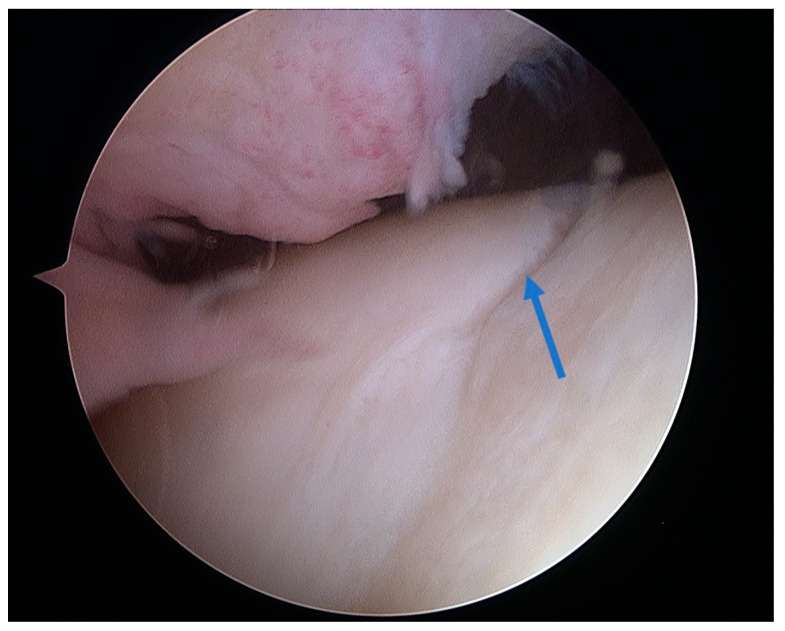
Arthroscopic view of grade III lesion with a visible cartilage deficit of less than 50% (arrow) in the femoral trochlear groove.

**Figure 6:**
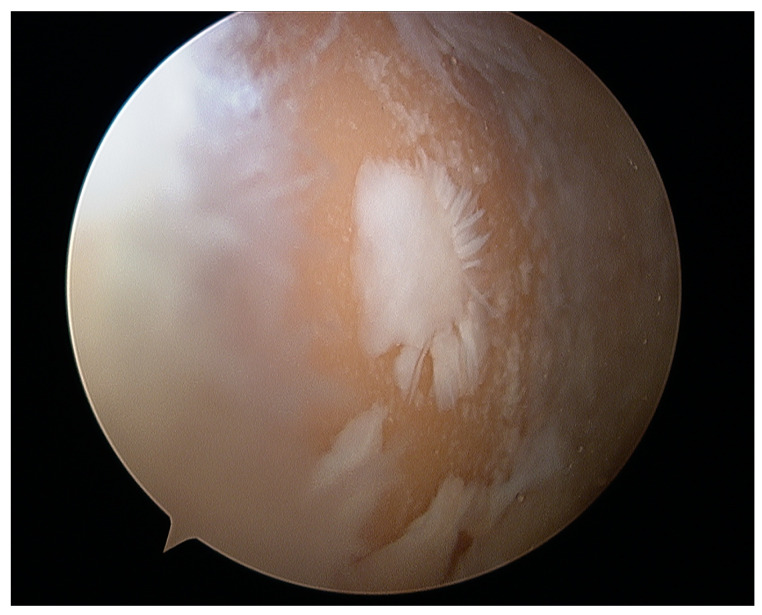
Arthroscopic view of grade IV lesion with subchondral bone exposure and complete cartilage loss, with only islands of cartilage visible on the medial femoral condyle.

At the macroscopic level, alterations in the composition of the cartilage matrix coincide with the emergence of surface fibrillations, which are characterized by the presence of microscopic cracks in the superficial zone. As OA advances, these cracks contribute to the detachment of cartilage fragments and the development of fissures that expand into the deeper layers of cartilage. Subsequently, the deep fissures within the affected cartilage cause its delamination, exposing the underlying zones of the calcified cartilage and subchondral bone [171,172,173]. Figure 3, Figure 4, Figure 5 and Figure 6 show the course of cartilage loss in a knee joint according to the ICRS [174] grading system. These changes include an increase in the volume, thickness, and outline of the cortical plate, as well as changes in bone mineralization and material characteristics. Additionally, OA is associated with changes in the architecture and mass of the subchondral trabecular bone, the development of bone cysts, and the presence of bone marrow lesions and osteophytes [175,176,177]. Subchondral bone cysts are frequently observed in individuals with advanced osteoarthritis. A concept has been developed that cysts are created within the subchondral bone, specifically at places where previous bone marrow lesions are present. This observation suggests that the development of cysts is directly linked to bone damage and necrosis, which, in turn, triggers the process of osteoclast-mediated bone resorption, ultimately resulting in cyst formation [178]. Osteophytes may potentially play a role in joint stabilization rather than actively contribute to the advancement of joint disease. Certainly, the elimination of osteophytes has been seen to result in increased joint instability in animal models of osteoarthritis [179]. Moreover, it is worth noting that no discernible correlation exists between the advancement of knee OA and the dimensions of osteophytes in human individuals with OA [180].

### 4.3. Synovial Fluid Changes

The synovium is a distinct type of connective tissue that serves as a lining for diarthrodial joints, envelops tendons, and constitutes the inner layer of bursae and fat pads. The synovium plays a crucial role in regulating the quantity and content of synovial fluid (SF), primarily through the synthesis of lubricin and hyaluronic acid. The synovium plays an important role in facilitating chondrocyte nourishment, together with the subchondral bone. This is particularly important because articular cartilage lacks its own vascular or lymphatic supply [181].

The synthesis and secretion of proteoglycan 4 (PRG4) protein, also known as lubricin, occur within articular joints, specifically articular chondrocytes [182] and synoviocytes [183] in the superficial zone. Lubricin is detected inside synovial fluid [184] and is also found at the surface of articular cartilage. Lubricin functions as a boundary lubricant, facilitating the reduction in friction during contact between the cartilage surfaces. In this context, lubrication is achieved through molecular interactions occurring at the surface. Additionally, it exhibits a synergistic effect with hyaluronan (HA) to further diminish friction to a level that is almost equal to that of complete synovial fluid [86]. HA levels are diminished in osteoarthritis compared to the healthy joint [185]. Similarly, subsets of people with OA exhibit a diminished lubricating capacity in relation to lubricin [186]. Kosinska et al. [187] quantified the levels of HA and lubricin in synovial fluid samples obtained from healthy joints, as well as from joints at different stages of osteoarthritis, including early-stage (eOA) and late-stage (lOA) osteoarthritis. The concentrations of HA were found to be the highest in the control SF, with a mean value of 2.2 mg/mL (range: 1.6–3.7 mg/mL). In comparison, the levels of HA in eOA SF were significantly lower, with a mean value of 1.7 mg/mL (range: 1.1–1.9 mg/mL). Similarly, the accumulation of HA in lOA SF were also lower, with a mean value of 1.9 mg/mL (range: 1.5–2.3 mg/mL), although this difference was not statistically significant. The levels of HA in eOA SF were 23.7% lower than those in the control SF, while the levels in lOA SF were 14.0% lower. The amount of lubricin in the control synovial fluid was measured to be 364.4 μg/mL (305.0–404.8 μg/mL). This concentration was found to be 1.5 times higher compared to the concentration of lubricin in the synovial fluid of individuals with early osteoarthritis (eOA), which was measured to be 244.5 μg/mL (119.6–381.7 μg/mL). Significantly, compared to the control synovial fluid, the content of lubricin in the synovial fluid for individuals with osteoarthritis decreased by 58.2% [152.3 μg/mL (108.2–183.9 μg/mL), *p* = 0.005]. The facilitation of low friction in the boundary mode and its potential impact on the shear deformation of cartilage is attributed to the lubrication of articular cartilage by synovial fluid. When conducting experiments on articular cartilage, researchers have observed that the presence of synovial fluid and its lubricant molecules can lead to reduced friction on the articular surface, thus demonstrating the effects of boundary lubrication [86,188]. The substitution of SF lubrication with phosphate-buffered saline (PBS) leads to an increase in boundary-mode friction [188].

Prior research has demonstrated that synovial fluid derived from human joints afflicted with OA exhibits typical lubricating properties [189]. Conversely, a reduction in the lubricating capacity of synovial fluid has been documented following several inflammatory and traumatic events, such as rheumatoid arthritis, [190] knee joint effusion after trauma, [189] meniscus removal, [191], and anterior cruciate ligament disruption [192]. A correlation has been discovered between a decrease in lubricin levels and an increase in friction inside the whole joint [108,190,192]. Teeple et al. [193] observed a marked decrease in the overall joint lubrication and an accompanying rise in friction persisting beyond the initial acute phase of the injury. The specific mechanisms underlying the deficit of lubricin remain unclear, although potential factors include reduced expression of lubricin by synoviocytes or superficial zone chondrocytes, depletion of these cells, and/or an elevated breakdown of lubricin. Mice lacking in lubricin exhibit clinical and radiographic manifestations of joint pathology as well as histological irregularities in their articulate joints that become more pronounced as they mature. The most significant characteristics include synovial hyperplasia and subintimal fibrosis, the presence of proteinaceous deposits on the surface of cartilage, irregularities in the cartilage surface and endochondral growth plates, as well as aberrant calcification observed in tendon sheaths and osteophytes [194]. The inclusion of lubricin into an in vitro bovine explant cartilage-on-cartilage-bearing system resulted in a considerable reduction in the coefficient of friction and chondrocyte death in the peripheral zone of cartilage. This finding confirmed the essential function played by lubricin in the prevention of cartilage degeneration [195]. The severity of both age-related and post-traumatic osteoarthritis was reduced in transgenic mice by the overproduction of lubricin. The observed decrease can be attributed to the inhibitory effect of lubricin on the expression of genes associated with cartilage breakdown and the enlargement of chondrocytes [192].

### 4.4. Mechanical Changes

Significant alterations in the functionality of cartilage are observed in individuals with osteoarthritis, leading to negative impacts on the weight-bearing, stabilizing, and lubricating capabilities of articular cartilage.

When subjected to tension, cartilage experiences a loading or stretching force, which causes collagen fibers and entangled proteoglycan molecules to align and elongate in the direction of the applied force. The primary source of resistance to tensile deformation and loads is mostly derived from the inherent stiffness of collagen fibrils [72,196,197]. The tensile modulus in the healthy human articular cartilage has been observed to range between 5 and 25 MPa. This variation depends on factors such as the specific position on the joint surface as well as the depth and orientation of the test specimen in relation to the joint surface [198,199]. The presence of osteoarthritis has been associated with a substantial reduction in the tensile modulus, with a potential loss of up to 90%. This decrease indicates a considerable level of damage to the solid network of cartilage [199]. Likewise, there have been documented reports of reduced tensile stiffness and fracture stress in the human cartilage affected by OA [196,198]. The changes are indicative of structural abnormalities in the collagen fibrillar network, as evidenced by both macroscopic and histological observations. The cartilage affected by degeneration also had a notably higher level of compliance to shear. This phenomenon was related to the presence of fibrillation on the articular surface and the depletion of the extracellular matrix [200]. In their study, Peters et al. [134] observed a significant reduction in the shear storage modulus by around 70–80% compared to the healthy condition.

Previous studies have demonstrated that the articular cartilage exhibiting surface fibrillation, pitting or fraying shows a higher level of compliance or deformability under compression [198,200,201]. Boschetti et al. [139] reported a reduction of 30% in the average thickness and a growth of 8% in the average water levels in OA samples compared to the healthy cartilage. These findings were consistent with the observations made in previous studies [100,156,202,203]. The mechanical properties of OA samples were also evaluated in comparison to those of the healthy cartilage. The static compressive modulus exhibited a decrease of 55–68%. Additionally, the permeability demonstrated an increase of 60–80%, while the dynamic compressive modulus experienced a decrease of 59–64%. Lastly, the static tension modulus displayed a decrease of 72–83% compared to the reference value. According to Armstrong and Mow [100], the compressive modulus of human cartilage tends to decrease as the severity of degeneration increases. Additionally, a reduction in the modulus was observed as individuals progressed in age. On the other hand, it was shown that neither age nor degeneration exhibited any significant variation in hydraulic permeability. Evidently, the OA-induced changes in cartilage, such as fibrillation, heightened hydration, and reduced proteoglycan content, would have a more significant impact on the inherent compressive stiffness of the cartilage compared to its flow-dependent behavior [204].

Ihnatouski et al. [114] observed that there was a decrease in the average values of the instantaneous modulus as the OA grade increased. The osteoarthritis-affected specimens were split into three groups: small, medium, and severely impacted. Young’s modulus for the normal cartilage ranged from 1.7 to 0.5 MPa, while the values for the three stages of OA wear were lower—1.14 to 1.3 MPa (small OA), 1.02 to 1.2 MPa (medium OA), and 0.82 to 1.2 MPa (severe OA). Additionally, atomic force microscopy surface-mapping was employed to examine the alterations in surface roughness that occur with an increase in OA stages. The results indicated a positive link between the two variables. Changes in the equilibrium modulus were also investigated. A study by Ebrahimi et al. [205] showed that the tibial plateaus exhibited a significant reduction in Eeq, reaching up to 80% compared to the healthy tissue. Similarly, Kleeman et al. [138] discovered that the Eeq of cartilage was reduced by around 40% from the early stages to the advanced stages of osteoarthritis.

In a study conducted by Huttu et al. [112], it was observed that mechanical parameters exhibited a negative correlation with cell volume. This relationship was attributed to an increase in the collagen orientation angle inside cartilage as osteoarthritis progressed. Additionally, Nissinen et al. [113] observed significant variations between early and advanced osteoarthritis, leading to a reduction in the initial modulus of the fibril network and the strain-dependent permeability. A positive relationship was observed between the total joint OA grade and the subchondral bone growth [134].

## 5. Mechanical and Frictional Features of Cartilage Repair Techniques—Are We Getting Close?

The intricate and dynamic nature of hyaline cartilage within the human body poses both challenges and opportunities in the realm of medical science. Cartilage plays a crucial role in maintaining joint function; however, its limited self-repair capability makes cartilage injuries a significant concern for surgeons and scientists. In this section, we will explore the principles, methodologies and outcomes of various cartilage repair techniques, ranging from simple interventions like microfractures to complex tissue-engineering constructs. A summary of the techniques mentioned below is given in Table 1.

### 5.1. Microfracture

**Figure 7:**
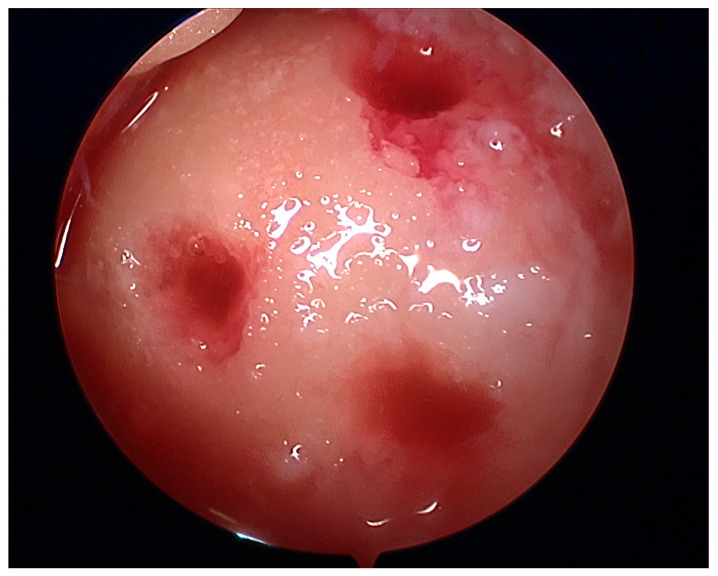
Arthroscopic view of microfractures on the medial femoral condyle. One can appreciate extravagation of bone marrow from the MFX site.

Full-thickness articular cartilage lesions hardly ever heal on their own [93,206]. Numerous techniques have been employed to activate bone marrow in the history of cartilage repair. A complete injury of the hyaline cartilage in a weight-bearing region between the femur and the tibia or in the patellofemoral joint is a common indication for microfracture. The indications for the microfracture technique are usually small lesions up to 2 cm^2^ without subchondral involvement. Exceptionally, it can be used for larger defects (>3 cm^2^) in less demanding patients [207]. The location of the lesions is also crucial, with much better results achieved at the femoral condyles than at the patellofemoral joint [208]. The microfracture (MFX) technique was extensively researched and developed by Steadman [209,210]. Over the years, advancements [25] have been made in the method, resulting in many improvements. These enhancements include removal of the calcified subchondral bone [211], establishment of straight and uniform cartilage margins [212] and execution of microfractures in close proximity to one another [213]. The perforation of the subchondral bone plate releases liquid bone marrow. Depending on the size of the awl/drill used for bone marrow stimulation, nanofractures with the use of 1 mm drills can be distinguished in this technique. Figure 7 shows the arthroscopic view of the medial femoral condyle with microfractures (MFX). The roughened surface produced by the surgeon provides an area to which the marrow clots can firmly adhere [209,214]. Mesenchymal stromal cells (MSCs) that are introduced into the damaged region have the ability to undergo differentiation into fibrochondrocytes. These fibrochondrocytes then proceed to occupy the defect and subsequently undergo remodeling, resulting in the formation of a fibrocartilage clot [215]. Nevertheless, the abundance of mesenchymal stromal cells is very limited and diminishes with an individual’s aging [216]. The composition of the clot primarily consists of type I collagen, which distinguishes it from the natural hyaline cartilage that predominantly comprises type II collagen [217]. Type II collagen possesses a higher concentration of hydroxylysine and a much greater amount of glycosylated hydroxylysine compared to type I collagen. These additional residues might confer distinctive physical characteristics onto type II fibrils [218]. Histological examinations of the tissue-healing process subsequent to microfracture surgery have revealed the predominant presence of fibrocartilage. In other cases, a hybrid repair tissue has been observed, characterized by varying levels of proteoglycan and type II collagen [219,220]. In contrast to hyaline cartilage, fibrocartilage has mechanical qualities that are less optimal for enduring the prolonged stresses associated with joint-loading, owing to its softer nature and lower capacity for tolerating shear stress [221]. The cells present in the fibrous tissue have an elongated phenotype resembling fibroblasts, both in terms of their physical form and the profile of genes they express. The matrix exhibits a reduced concentration of glycosaminoglycans and a higher presence of type I collagen [222,223]. Ebenstein et al. [224] reported that fibrous repair cartilage exhibited a contact stiffness of 0.03 ± 0.01 kN m^−1^, which was approximately one order of magnitude lower than the contact stiffness of the healthy cartilage (0.17 ± 0.039 kN m^−1^). The lower compression stiffness could explain the lower resilience of the fibrous repair cartilage to mechanical load [225].

The microfracture technique is frequently used for the treatment of chondral injuries. Nevertheless, like any other surgical procedure, it poses a distinct set of possible risks. Incomplete adhesion or partial filling of the defect by unstable blood clots may lead to poor healing [214]. The occurrence of osseous outgrowth has been observed subsequent to inadvertent removal of the subchondral bone during the operation. Osseous overgrowth occurs frequently, with a reported incidence ranging from 25% to 49% among patients [226,227].

Good short-term clinical results have been reported for the treatment of cartilage lesions using the microfracture technique [228]. However, a longer follow-up indicated steadily decreasing satisfaction with the results and lower durability of the repair over the years [229,230]. Orth et al. [231] in their systematic review reported a failure of rate 11–27% in 5 years of observation and 6–32% during a 10-year period.

### 5.2. Autologous Matrix-Induced Chondrogenesis (AMIC)

Autologous Matrix-Induced Chondrogenesis (AMIC) is a single-stage procedure for cartilage repair combining microfractures and application of external scaffold. One of the reasons for failure of isolated microfractures may be the lack of protection of the repair site and washing out of MSCs [232]. Adding a matrix allows for stabilization of the clot and provides a scaffold for bone marrow cells, facilitating their differentiation towards the cartilage lineage [233]. Longer follow-up of this technique shows promising results. After 2 years, the outcomes are comparable to isolated microfractures, but after this time, the microfractures are characterized by a decrease in satisfactory results, in contrast to the AMIC technique, which maintains its functional parameters for up to 5 years [230].

### 5.3. Osteochondral Autograft Transfer System (OATS)

**Figure 8:**
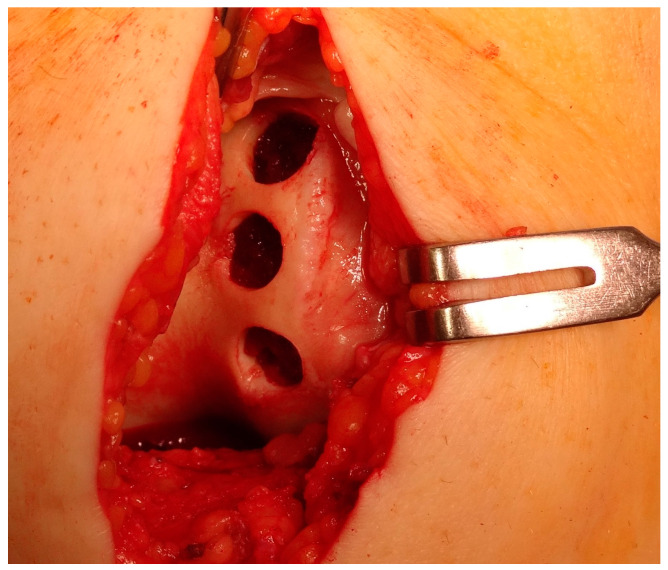
Intraoperative view of donor site preparation for osteochondral blocks implantation on the lateral femoral condyle.

**Figure 9:**
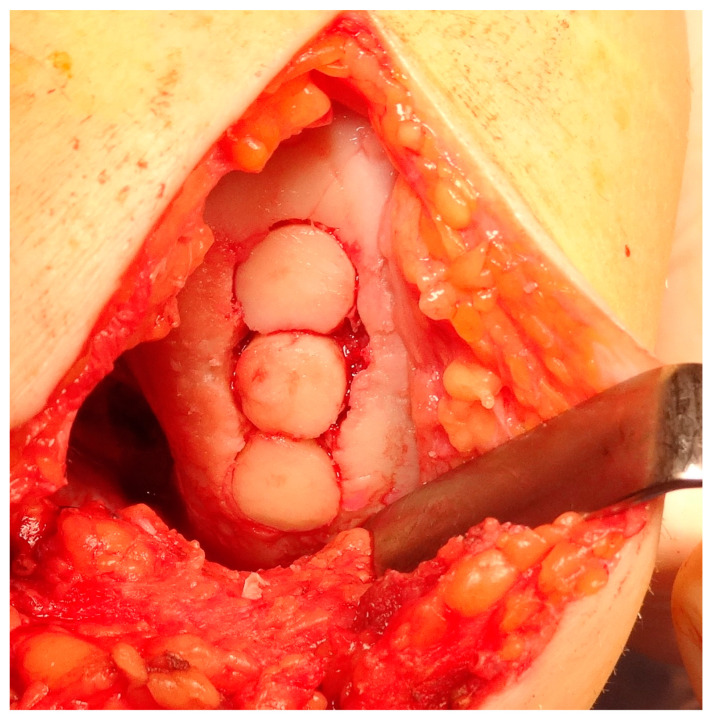
Intraoperative view of the final result with complete cartilage loss area coverage.

Osteochondral autograft transplantation (OAT) involves the transplantation of grafts obtained from the non-weight-bearing areas of the joint to the injured regions that bear more weight [234]. The application of autograft results in a more expedited and dependable process of osseous integration compared to the osteochondral allograft. Furthermore, the autograft presents several advantages, including convenient accessibility to donor cartilage, capacity for addressing lesions of different sizes, and utilization of the native hyaline cartilage containing functional and fully developed chondrocytes [235,236]. Good results of OATS have been described for small defects (<2 cm^2^), but larger defects ranging from 2 to 4 cm^2^ can also be treated beneficially with this method, especially in young, demanding patients [237]. This technique is the first line of treatment for cartilage lesions involving the subchondral layer [235]. The histologic examination of the transplanted osteochondral graft has revealed that in an ideal OATS, the grafts are successfully integrated into the defects to preserve the structural integrity of the hyaline cartilage and cancellous bone. This integration also ensures the maintenance of a smooth and congruent articular surface in the weight-bearing regions [238,239]. Intraoperative views of the OATS procedure are shown in Figure 8 and Figure 9.

Nakaji et al. [240] provided a comprehensive analysis of the progressive alterations in the structural characteristics of an osteochondral cylinder graft–recipient construct. The primary focus of this study was to evaluate the stiffness of articular cartilage using a rabbit model. The articular cartilage stiffness of the osteochondral transplant was within normal parameters upon its first placement (107,695.1 ± 11,610.1 N/m^2^). During the first, third, and eight weeks following the surgical procedure, it was noticed that the stiffness levels decreased (95,386.8 ± 2689.4, 92,899.3 ± 3748.2, and 95,969.8 ± 2157.1 N/m^2^, respectively) compared to the stiffness typically detected in healthy cartilage (100,027.5 ± 396.4 N/m^2^). Additionally, the histological analysis revealed an increase in the bone trabeculae inside the subchondral region. At the 12-week post-operative mark, the articular cartilage of the osteochondral graft exhibited normal stiffness (104,683.7 ± 3311.5 N/m^2^), and the bone trabeculae in the subchondral region demonstrated effective remodeling.

A study by Kuroki et al. [241] investigated the mechanical impact of an OATS on articular cartilage in a porcine model. They employed an ultrasonic measurement system to assess the immediate post-surgical outcomes. The findings of the study indicated that the surgical procedure of osteochondral grafting did not induce any significant alterations in the stiffness (9.2 ± 1.78 and 9.0 ± 1.91 [corresponding values, mean ± SD] before harvesting and after grafting, respectively, in a 6 mm-plug model and 5.8 ± 1.54 and 5.8 ± 1.87, respectively, in a 5 mm-plug model), surface irregularity (0.7 ± 0.10 μ seconds and 0.7 ± 0.11 μ seconds, before harvesting and after grafting, respectively, in a 6 mm-plug and 0.8 ± 0.12 μ seconds and 0.8 ± 0.09 μ seconds, respectively, in a 5 mm-plug model), or thickness of the graft plug (2.7 ± 0.57 μ seconds and 2.7 ± 0.62 μ seconds, before harvesting and after grafting, respectively, in a 6 mm-plug model and 2.4 ± 0.81 m seconds and 2.3 ± 0.67 m seconds, respectively, in a 5 mm-plug model). Additionally, it was hypothesized that in the event of mechanical alterations, a change in stiffness would be more likely related to the healing or remodeling process rather than to the surgical procedure.

Lane et al. [242] examined the biochemical and biomechanical alterations throughout a goat osteochondral autograft model after 12 weeks following surgery. The stiffness of the healthy cartilage was 0.79 ± 0.15 N/mm, whereas the cartilage of the transferred plugs ranged 5.29 ± 1.04 N/mm. This indicated that the graft cartilage had a stiffness that was 6 to 7 times larger than that of the control normal tissue. Moreover, viability of the cells in the bone plugs were examined by confocal microscopy. In total, 95% of the cells counted manually were viable 12 weeks after grafting. The assessment of the joint surfaces showed no significant degenerative changes at either the recipient or the donor locations 12 weeks post implantation.

The biomechanical and histological characteristics of the OATS were also investigated by Nam et al. [243] in a rabbit model. The stiffness of the 12-week grafts (1213.6 ± 309.0 N/mm) was found to be substantially greater than that of the 6-week grafts (483.1 ± 229.1 N/mm) and natural cartilage (774.8 ± 117.1 N/mm). The stiffness of the grafts at the 6-week mark revealed a statistically significant reduction in comparison to the stiffness observed in the natural cartilage. Furthermore, with regard to a potential score of 24.0 points, the average values for the overall healing indices (Modified O’Driscoll Histological Score) [244] were as follows: 6-week OAT, 21.6 ± 1.3; 12-week OAT, 21.0 ± 1.8; 6-week full-thickness defects, 11.5 ± 2.8; and 12-week full-thickness defects, 10.8 ± 4.4. The histology scores of the OAT groups were considerably superior compared to the full-thickness defects groups in both time periods.

The successful use of autograft is subject to some constraints, with defect size being the primary one. Lesions above 3 cm^2^ in size are susceptible to experiencing symptomatic donor–site morbidity, resulting in pain and associated symptoms [245]. The rate of donor–site morbidity has been reported to range from 2.3% to 12.6% [246,247]. However, OATS has very good long-term clinical results with functional benefits and survival beyond 15 years [248].

### 5.4. Autologous Chondrocyte Implantation—ACI

The Autologous Chondrocyte Implantation (ACI) procedure was first introduced in 1994 [206]. It involves a two-step approach, beginning with the collection of a sample of the patient’s articular cartilage in the first stage. Subsequently, after ex vivo multiplication, the cells are inserted to the chondral defect during the second stage. The ACI possesses the notable benefit of effectively addressing extensive lesions measuring up to 10 cm^2^ by the restoration of cartilage that closely resembles hyaline cartilage [249,250,251,252,253]. The growth of hyaline-like tissue during the healing of chondral lesions is expected to yield biomechanical qualities that are comparable to those of the healthy cartilage, as shown by stiffness measures [251]. The grafted area’s stiffness was measured to be 2.4 ± 0.3 N, while the normal cartilage’s result was 3.2 ± 0.3 N. Moreover, the average stiffness measurement in the grafted sections containing hyaline tissue was found to be 3.0 ± 1.1, whereas a stiffness of 1.5 ± 0.35 was reported in the repairs involving fibrous tissue. In 8 out of 12 cases of stiffness testing, the indentation measurement exhibited a value that was equal to or more than 90% of the value seen in the healthy cartilage.

According to Vasara et al. [253], the stiffness of the repaired tissue exhibited a notable increase, reaching 62% of the stiffness observed in the surrounding cartilage. The indentation force of the repair tissue in six individuals was shown to exceed 80% of the adjacent cartilage, indicating a potential presence of hyaline-like repair. The mean indentation force of the repair tissue was 2.04 ± 0.83 N, which accounted for 62% of the adjacent cartilage (3.58 ± 1.04 N). However, it is important to note that there was a significant difference in the stiffness of the repair tissue.

Henderson et al. [254] conducted an evaluation of 66 ACI repairs for articular cartilage injuries. The mean normalized stiffness for the entire sample of 66 lesions was found to be 104% at an average follow-up period of 22.1 months post-implantation. A notable observation was made regarding the stiffness of both hyaline articular cartilage and hyaline-like repairs, with about half of these samples exhibiting greater stiffness compared to the adjacent cartilage. It was suggested that it could be due to the difference in matrix composition during the healing process. The alteration in cartilage matrix composition occurring with the aging process may differ from the repair composition that more closely approaches the stiffer structure often observed in children. On the other hand, it is possible that a higher repair stiffness would lead to the occurrence of symptomatic repair due to abnormal load transmission, which is comparable to the reported high stiffness of the subchondral bone plate in individuals with OA. Hence, the increased stiffness might have negative implications for joint functionality. Moreover, based on the clinical and arthroscopic observations, repairs were categorized into two groups: ACI-unrelated problems (Group A) and ACI-related problems (Group B). In Group A, a majority of repairs, namely 65%, consisted of either hyaline or hyaline-like cartilage, but in Group B this proportion was significantly lower and amounted to 28%. Autologous chondrocyte repairs consisting of fibrocartilage had a higher prevalence of morphologic defects and manifested symptoms at an earlier stage compared to the repairs using hyaline or hyaline-like cartilage. The reparative characteristics of hyaline articular cartilage were shown to have biomechanical parameters that were equivalent to the adjacent cartilage and higher than those observed for fibrocartilage repairs.

Despite initial good clinical results, ACI is characterized by a higher rate of complications, such as periosteal patch hypertrophy, high reoperation rates, bulky sutures, and cell leakage [255]. The occurrence of such adverse effects paved the way for the matrix-based modification of this technique using matrix-induced autologous chondrocyte implantation (MACI).

### 5.5. Matrix-Induced Autologous Chondrocyte Implantation—MACI

**Figure 10:**
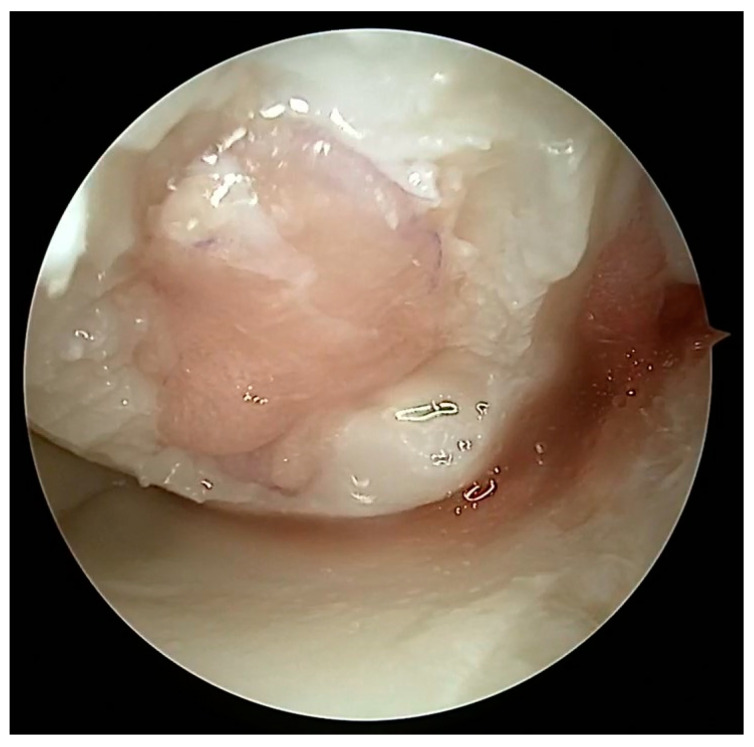
Dry arthroscopy view of a biodegradable non-woven hyaluronic acid scaffold covering a cartilage defect on the medial femoral condyle.

MACI is a more recent variation of ACI, which incorporates a collagen scaffold to facilitate the use of autologous cells and promote directed tissue regeneration. This next-generation method has the advantage of utilizing the patient’s own cells while employing a biocompatible scaffold made of collagen. A surgical visualization via a dry arthroscopy technique is shown in Figure 10. The MACI implant possesses intrinsic benefits such as the ability to be surgically implanted by arthroscopy or miniarthrotomy, no periosteal harvest, and its utilization of tissue adhesive as a substitute for sutures [256]. The efficacy of MACI has been assessed in many animal experiments, demonstrating its ability to enhance the healing process in full-thickness cartilage injuries. The immunological or inflammatory responses elicited by the membrane alone have been assessed and shown to be modest [257,258]. MACI is indicated as a first line of treatment for lesions above 2 cm^2^ and second for defects below 2 cm^2^ [259].

A study by Lee et al. [260] demonstrated that the use of a MACI graft in combination with a type II collagen membrane resulted in an aggregate modulus that was 15% of the modulus observed in the native tissue. The results of stiffness tests conducted on an ovine model demonstrated that the MACI grafts exhibited a stiffness range of 16% to 50% in comparison to the natural cartilage [257,261].

Griffin et al. [262] investigated the mechanical characteristics of MACI cartilage repair in an equine model. The findings indicated that the compressive and frictional properties of the repaired tissue were comparable to those of the natural tissue. The equilibrium modulus of the cartilage obtained from the defects that underwent MACI was found to be 70% of that observed in the normal cartilage. This value was not found to have a statistically significant difference when compared to the equilibrium modulus of the native control tissue. Moreover, there was no statistically significant difference between the control tissue and the implant groups in terms of the average values of boundary mode friction coefficients which varied from 0.42 to 0.52. The shear modulus values for the healthy cartilage generally varied between 1.0 and 1.5 MPa; however, the shear moduli of the cartilage from all categories of injuries were much lower, ranging from 0.2 to 0.5 MPa, which represents a reduction of 4 to 10 times compared to the healthy cartilage. The low shear modulus of the grafts made the restored cartilage vulnerable to mechanical failure or deterioration.

Schuette et al. [263] reported favorable mid- to long-term clinical results of the MACI technique. It significantly increased patient-related scores (KOOS, SF-36, Tegner). Comparison of MFx and MACI at 5-year follow-up shows significantly better results in the KOOS pain score and functional scales and non-significantly lower risk of failure [206].

### 5.6. Tissue Engineering

**Figure 11:**
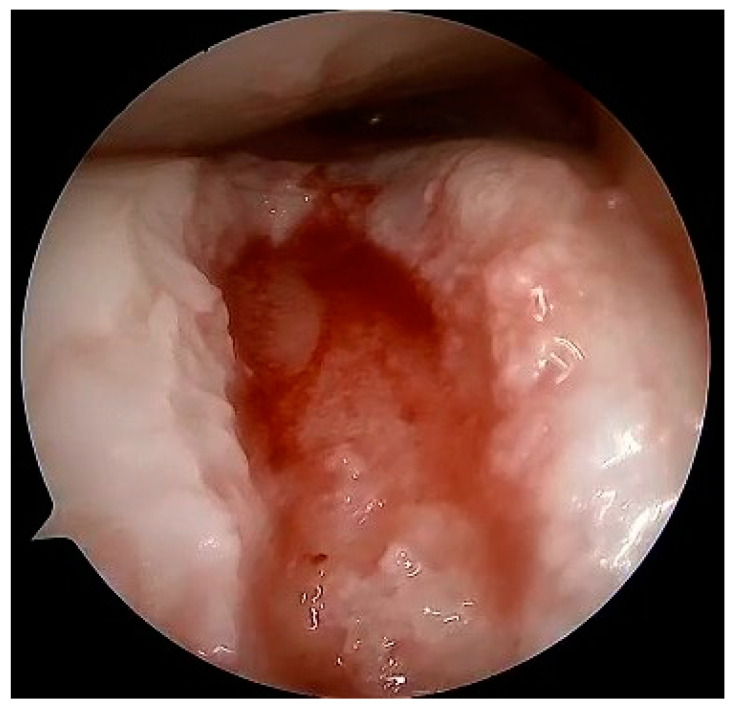
Dry arthroscopy view of a full-thickness cartilage injury in the femoral trochlea after debridement and preparation for matrix implantation.

**Figure 12:**
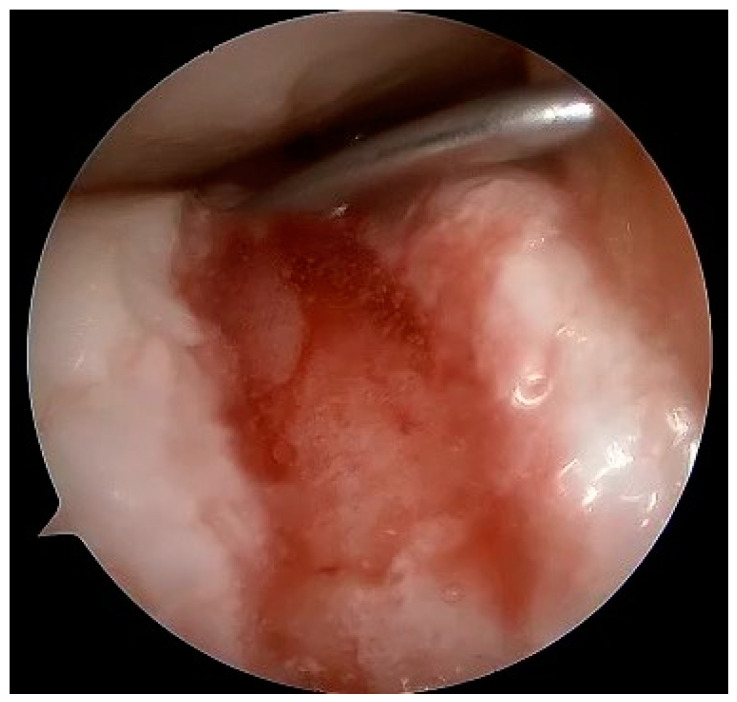
Dry arthroscopy view of the same defect filled with cell-free matrix gel.

The concept of osteochondral and cartilage tissue engineering has emerged as a means to forward the development of novel and enhanced therapeutic interventions. There exist two primary approaches to the restoration of impaired osteochondral modulus and complete-thickness cartilage through the use of tissue-engineering techniques. The objective is to create artificial cartilage structures that replicate the structural characteristics, mechanical attributes and, therefore, biological functionalities of natural cartilage tissues. An alternative approach puts greater emphasis on the field of regenerative medicine. The fundamental idea revolves around the administration of suitable biomaterials in the form of artificial extracellular matrix to stimulate cellular growth, proliferation, and differentiation at the injury zone. This approach relies on the inherent biological processes involving cellular interactions and biomolecules to facilitate the regeneration of articular cartilage and subchondral bone [264]. An implantation of the collagen matrix with a preparation of the donor site is shown in Figure 11 and Figure 12.

Koh et al. [265] utilized the finite element analysis to demonstrate the efficacy of a scaffold with ideal mechanical qualities in promoting cartilage regeneration within a cartilage defect. It was assumed that the implantation of a scaffold with ideal mechanical qualities would serve to mitigate cell death and promote increased development of cartilage tissue.

The scaffolds utilized in cartilage tissue engineering are mostly composed of carbohydrates such as alginate, chitosan, poly-L-lactide/poly(glycolic acid) (PLLA/PGA), agarose, and hyaluronic acid, as well as proteins like collagen and gelatin. Growth factors are employed to induce the proliferation and differentiation of cells that will be transplanted onto the scaffold, hence promoting the maintenance of their chondrogenic phenotype [266]. Other authors have explored alternative mechanical and chemical stimuli in pre-implantation culture in order to enhance lubrication efficiency [267]. Scaffolds which are better-structured present higher elastic values [268].

Hydrogels are polymer networks that exhibit a three-dimensional structure characterized by significant swelling and porosity at the molecular level. This unique structure enables the transport of diverse solutes and nutrients inside the hydrogel matrix. The fabricated structure possesses cell compatibility, enabling the containment of various cell types such as chondrocytes and stromal cells. Moreover, a variety of hydrogel characteristics may be adjusted to enhance their effectiveness in tissue regeneration. These parameters encompass polymer chemistry, crosslinking density, degradation, mechanical qualities, and release kinetics of biological components [269]. Hydrogels with reduced crosslinking density exhibit lower mechanical properties. Dynamic loading conditions have the potential to promote the process of chondrogenesis in mesenchymal stromal cells [269,270]. The mechanical strength of natural hydrogel scaffolds can vary between 0.45 and 5.65 MPa, but synthetic hydrogels have the potential to reach values ranging from 15 to 125 MPa [271].

When compared to solid scaffolds, hydrogels facilitate the adoption of a more spherical shape by cells, which is a hallmark feature of the chondrogenic phenotype. This, in turn, leads to a reduction in the production of fibrous tissues [272]. However, hydrogels have restricted mechanical characteristics, which renders them susceptible to failure. This drawback poses a significant disadvantage, particularly considering that articular cartilage is exposed to substantial mechanical pressures. Furthermore, it should be noted that these particular materials still exhibit limitations in their capacity for effective integration with the adjacent tissues [273].

Also worth highlighting in this paper is the need for adequate oxygen concentration during chondrocyte culturing [274,275,276]. It has been proven that a physiological (5%) oxygen concentration while culturing chondrocytes promotes proper and adequate chondrogenesis. An optimal oxygen concentration increases GAG production, therefore increasing compressive strength of the constructs [277]. Physioxia during chondrocyte culturing also promotes collagen type II production [278].

**Table 1:** Comparison of the biomechanical properties of different cartilage repair methods.

Study	Operative Technique	Tissue Type	Measurement System	Biomechanical Results	Additional Findings
Ebenstein et al. [224]	MFx	Animal model—rabbits	Nanoindentation	Stiffness 0.03 ± 0.01 kN m^−1^—fibrous repair 0.17 ± 0.039 kN m^−1^—normal cartilage	
Nakaji et al. [240]	OATS	Animal model—rabbits	Tactile frequency	Stiffness 107,695.1 ± 11,610.1 N/m^2^ after procedure; 95,386.8 ± 2689.4 after 1 week; 92,899.3 ± 3748.2 after 3 weeks; 95,969.8 ± 2157.1 N/m^2^ after 8 weeks; 104,683.7 ± 3311.5 N/m^2^ after 12 weeks 100,027.5 ± 396.4 N/m^2^—normal cartilage	Increase in bone trabeculae inside the subchondral region.
Kuroki et al. [241]	OATS	Animal model—porcine	Ultrasonic	Stiffness 9.2 ± 1.78 (before harvesting) and 9.0 ± 1.91 (after grafting) [corresponding values]—6 mm plug model 5.8 ± 1.54 (before harvesting) and 5.8 ± 1.87 (after grafting)—5 mm plug model	Changes in stiffness are more likely related to the healing or remodeling process rather than to the surgical procedure
Lane et al. [242]	OATS	Animal model—goats	Indentation	Stiffness 5.29 ± 1.04 N/mm—transferred plugs after 12 weeks 0.79 ± 0.15 N/mm—normal cartilage	In total, 95% of the cells counted manually by confocal microscopy were viable 12 weeks after transfer
Nam et al. [243]	OATS	Animal model—rabbits	Indentation	Stiffness 1213.6 ± 309.0 N/mm—12 weeks after transplant 483.1 ± 229.1 N/mm—6 weeks after transplant 774.8 ± 117.1 N/mm—normal cartilage	Modified O’Driscoll Histological Score (24 points max): 6-week OAT, 21.6 ± 1.3; 12-week OAT, 21.0 ± 1.8; 6-week full-thickness defects, 11.5 ± 2.8; 12-week full-thickness defects, 10.8 ± 4.4
Hangody et al. [238]	OATS	Human	Arthroscopic Indentation	Stiffness Stiffness of the resurfaced area similar to the surrounding hyaline cartilage (no numerical data)	
Peterson et al. [251]	ACI	Human	Indentation	Stiffness 2.4 ± 0.3 N—grafted area 3.2 ± 0.3 N—normal cartilage 3.0 ± 1.1 N—mean value at hyaline repair 1.5 ± 0.35—mean value at fibrous repair	
Vasara et al. [253]	ACI	Human	Indentation	Stiffness 2.04 ± 0.83 N—repair tissue 3.58 ± 1.04 N—normal cartilage	
Henderson et al. [254]	ACI	Human	Arthroscopic Indentation	Stiffness 3 ± 1.5 N—maximum stiffness of the repair Avg. normalized stiffness—104% of the surrounding hyaline cartilage	
Lee et al. [260]	MACI	Animal model—canine	Indentation	Aggregate modulus 15% of the native cartilage	
Griffin et al. [262]	MACI	Animal model—equine	Custom tribometer	Avg. values of boundary mode friction coefficients—from 0.42 to 0.52 Shear modulus 0.2 to 0.5 MPa—repaired cartilage 1.0 to 1.5 MPa—normal cartilage	
Franke et al. [221]	MACI (periosteal cells)	Animal model—miniature pigs	Nanoindentation	Stiffness 0.5 kN m^−1^—repaired cartilage 4.0 kN m^−1^—normal cartilage	
Tanaka et al. [268]	Tissue Engineering	PLLA scaffolds	Indentation	Elastic moduli 3000–28,000 kPa	

## 6. Conclusions

As shown in this review, the articular cartilage is one of the most sophisticated tissues in human body. Although it can withstand various forces without harm, once injured, it gives rise to the development of osteoarthritis due to ECM breakdown, resulting in increased load transmitted to the subchondral bone. This review has also demonstrated the impact of various mechanical and tribological properties on cartilage deterioration and repair techniques. Out of the mentioned repair techniques, OATS showed the closest resemblance to the native hyaline cartilage with regard to mechanical properties. This is attributed to the healthy subchondral bone, which is implemented with the overlying cartilage. Cartilage repair techniques which do not utilize subchondral bone, such as ACI and MACI, showed a decrease in mechanical properties, especially in shear modulus and stiffness. On the other hand, these are techniques which do not require harvesting of high-volume healthy cartilage to fill the defect. Up to date, there is no strong consensus regarding cartilage repair methods and mechanical testing. Therefore, more studies are required to find the best solution for the patient. In future, not only mechanical properties but also signaling pathways and inflammation modulation will play a role in finding the best solution for cartilage repair.

## Data Availability

Not applicable.
