# Correction: Krakowski et al. Cartilage Integrity: A Review of Mechanical and Frictional Properties and Repair Approaches in Osteoarthritis. *Healthcare* 2024, *12*, 1648

**DOI:** 10.3390/healthcare13233173

**Published:** 2025-12-04

**Authors:** Przemysław Krakowski, Adrian Rejniak, Jakub Sobczyk, Robert Karpiński

**Affiliations:** 1 Department of Trauma Surgery and Emergency Medicine, Medical University, 20-059 Lublin, Poland; 2 Orthopaedic and Sports Traumatology Department, Carolina Medical Center, Pory 78, 02-757 Warsaw, Poland; adrian.rejniak@carolina.pl (A.R.); jakub.sobczyk@carolina.pl (J.S.); 3 Department of Machine Design and Mechatronics, Faculty of Mechanical Engineering, University of Technology, 20-618 Lublin, Poland; 4 Department of Psychiatry, Psychotherapy and Early Intervention, Medical University, 20-059 Lublin, Poland

**Text Correction**

There was an error in the original publication [1]. Following an internal review, and in line with editorial recommendations and concerns raised by the authors of a previously published review [2], it was found that Subsections 3.2 (Tensile and Shear Properties of Cartilage) and 3.3 (Tribological Properties of Cartilage) in our manuscript contained textual similarities to the review by Belluzzi et al. [2]. Although these sections were paraphrased, they closely followed the organization, commentary style, and reference sequencing of the work by Belluzzi et al. [2].

This similarity concerned multiple parts of the text, including the descriptions of atomic force microscopy, indentation, compression, and friction tests, as well as the associated interpretation of data and choice of references. While our intention was to provide a broad literature overview, we acknowledge that the original version of our manuscript did not cite the review by Belluzzi et al. [2], despite exhibiting a significant degree of textual and structural alignment with that work. This omission was entirely unintentional, and we regret that it was not identified prior to publication. 

To ensure clarity, transparency, and adherence to best publication practices, Sections 3.2 and 3.3 have been entirely removed. In their place, we have included a concise summary of the topic along with an explicit reference to the work by Belluzzi et al. [2], which contains a comprehensive and authoritative discussion of the respective techniques and findings:

*3.2. Summary of Tensile, Shear, and Tribological Properties of Articular Cartilage*

The tensile, shear, and tribological properties of articular cartilage constitute essential determinants of its mechanical behavior under both physiological and pathological conditions. These characteristics are of particular importance in the context of osteoarthritis (OA), wherein progressive structural and biochemical degradation impairs the tissue’s ability to distribute mechanical loads, increases interfacial friction, and reduces resistance to shear-induced deformation [110–115].

When subjected to compressive loading, articular cartilage exhibits tensile strain and viscoelastic responses, primarily governed by the intricate interactions between the collagen fiber network and the proteoglycan-rich extracellular matrix. Resistance to shear stress is predominantly provided by the solid matrix and is highly dependent on the zonal organization of collagen architecture and proteoglycan concentration. Both tensile and shear moduli vary as a function of cartilage depth, the methodology employed for mechanical evaluation, and the stage of degenerative change. On a macroscopic scale, these mechanical parameters correlate strongly with the orientation of collagen fibers and the tissue’s biochemical composition [116–123].

Tribological characteristics, including the coefficient of friction (COF), are influenced by several factors such as lubrication regime, the biochemical composition of synovial fluid, surface morphology, and the degree of tissue hydration. Healthy articular cartilage demonstrates remarkably low COF values (down to 0.002), primarily attributable to mechanisms of interstitial fluid pressurization and boundary lubrication mediated by molecular constituents such as lubricin and hyaluronic acid [124–126]. In osteoarthritic cartilage, the coefficient of friction is elevated, typically as a consequence of glycosaminoglycan depletion, surface fibrillation, and diminished synthesis of endogenous lubricants [127–134].

A wide array of experimental methodologies has been applied to characterize these biomechanical properties, including atomic force microscopy (AFM) [131–133], indentation testing [135–138], compression tests [139–142], tensile tests [143–147], and tribological evaluation. Each technique yields method-specific values, which may differ based on the measurement scale, applied loading conditions, and sample preparation protocols. While AFM enables nanoscale assessment of surface mechanical properties, macro-scale tribological tests are designed to replicate physiologically relevant joint loading and lubrication environments [148–151].

A comprehensive and detailed description of experimental methodologies, numerical findings, and interpretative frameworks related to cartilage mechanics is available in the review by Belluzzi et al. [152]. That work offers a rigorous analysis of depth-dependent variations in mechanical moduli, protocol-specific test outcomes, and limitations inherent to various measurement approaches, providing a valuable reference for readers seeking in-depth technical insight.

We sincerely apologize to the readers, reviewers, and the editorial board for this oversight and any confusion it may have caused. We also extend our sincere thanks to the complainants for raising the issue and apologize for the insufficient attribution of their work. We remain fully committed to maintaining the highest standards of scientific integrity, transparency, and ethical publishing.

****

**References**

With this correction, the order of some references have been adjusted accordingly. 

The authors state that the scientific conclusions are unaffected. This correction was approved by the Academic Editor. The original publication has also been updated.
